# INPP4B promotes cell survival via SGK3 activation in NPM1-mutated leukemia

**DOI:** 10.1186/s13046-018-0675-9

**Published:** 2018-01-17

**Authors:** Hongjun Jin, Liyuan Yang, Lu Wang, Zailin Yang, Qian Zhan, Yao Tao, Qin Zou, Yuting Tang, Jingrong Xian, Shuaishuai Zhang, Yipei Jing, Ling Zhang

**Affiliations:** 1 Key Laboratory of Laboratory Medical Diagnostics Designated by the Ministry of Education, School of Laboratory Medicine, Chongqing Medical University, Chongqing 400016, China. No.1, Yixueyuan Road, Chongqing, 400016 China; 2 Center for Hematology, Southwest Hospital, Third Military Medical University, Chongqing, China; 3 The Center for Clinical Molecular Medical detection, The First Affiliated Hospital of Chongqing Medical University, Chongqing, China

**Keywords:** Acute myeloid leukemia, Nucleophosmin, Gene mutation, INPP4B, SGK3, AKT, Ets-1, Cell survival

## Abstract

**Background:**

Acute myeloid leukemia (AML) with mutated nucleophosmin (NPM1) has been recognized as a distinct leukemia entity in the 2016 World Health Organization (WHO) classification. The genetic events underlying oncogenesis in NPM1-mutated AML that is characterized by a normal karyotype remain unclear. Inositol polyphosphate 4-phosphatase type II (INPP4B), a new factor in the phosphoinositide-3 kinase (PI3K) pathway-associated cancers, has been recently found a clinically relevant role in AML. However, little is known about the specific mechanistic function of INPP4B in NPM1-mutated AML.

**Methods:**

The INPP4B expression levels in NPM1-mutated AML primary blasts and AML OCI-AML3 cell lines were determined by qRT-PCR and western blotting. The effect of INPP4B knockdown on OCI-AML3 leukemia cell proliferation was evaluated, using the Cell Counting Kit-8 and colony formation assay. After INPP4B overexpression or knockdown, the activation of serum and glucocorticoid-regulated kinase 3 (SGK3) and AKT was assessed. The effects of PI3K signaling pathway inhibitors on the levels of p-SGK3 in OCI-AML3 cells were tested. The mass of PI (3,4) P_2_ and PI (3) P was analyzed by ELISA upon INPP4B overexpression. Knockdown of SGK3 by RNA interference and a rescue assay were performed to confirm the critical role of SGK3 in INPP4B-mediated cell survival. In addition, the molecular mechanism underlying INPP4B expression in NPM1-mutated leukemia cells was explored. Finally, Kaplan–Meier survival analysis was conducted on the NPM1-mutated AML cohort stratified into quartiles for INPP4B expression in The Cancer Genome Atlas (TCGA) dataset.

**Results:**

High expression of INPP4B was observed in NPM1-mutated AML. Knockdown of INPP4B repressed cell proliferation in OCI-AML3 cells, whereas recovered INPP4B rescued this inhibitory effect in vitro. Mechanically, INPP4B enhanced phosphorylated SGK3 (p-SGK3) status, but did not affect AKT activation. SGK3 was required for INPP4B-induced cell proliferation in OCI-AML3 cells. High levels of INPP4B were at least partially caused by the NPM1 mutant via ERK/Ets-1 signaling. Finally, high expression of INPP4B showed a trend towards lower overall survival and event-free survival in NPM1-mutated AML patients.

**Conclusions:**

Our results indicate that INPP4B promotes leukemia cell survival via SGK3 activation, and INPP4B might be a potential target in the treatment of NPM1-mutated AML.

## Background

Acute myeloid leukemia (AML) is an aggressive bone marrow malignancy that arises from the somatic transformation of hematopoietic stem and progenitor cells [1]. Cytogenetic analysis assigns about 50% of AML patients to the normal karyotype (NK-AML) [2–4]. The discovery of numerous genetic alterations that are associated with NK-AML through whole-genome analyses has led to significant advances in AML research [5]. Nucleophosmin (*NPM1*) is one of the most frequently altered genes in NK-AML, accounting for one-third of AML cases (50 to 60% of adult NK-AML) [6]. To date, > 55 unique mutations have been identified in exon 12 of the gene *NPM1*, and the most common mutation is referred to as the type A mutation (NPM1-mA) with a 4-base-pair TCTG insertion at nucleotide position 960 [7, 8]. Because of its distinctive molecular and clinical characteristics, NPM1-mutated AML has been defined as a distinct entity in the 2016 updated World Health Organization (WHO) classification of myeloid neoplasms [9, 10]. Although the NPM1 mutation is an AML-driving lesion, this mutation alone is not sufficient to cause AML and it requires a cooperative event that aids leukemogenesis [11].

An accumulating body of evidence indicates that the phosphoinositide-3 kinase (PI3K) pathway plays an important role in the regulation of hematopoiesis [12]. Abnormal activation of the PI3K signaling pathway has been reported in >50% of AML cases [13]. Activated PI3K phosphorylates the phosphatidyl-inositol bisphosphate (PIP_2_) to generate phosphatidyl-inositol trisphosphate (PIP_3_), and thereby facilitates recruitment and activation of the AKT protein [14, 15]. In turn, PI3K/AKT signaling can be terminated by several phosphoinositide phosphatases that dephosphorylate phosphatidylinositol phosphate (PIP) species, i.e., the phosphatase and tensin homolog (PTEN) and src homology 2-containing inositol phosphatase (SHIP) family hydrolyze PIP_3_ to generate PIP_2_ [16, 17]. Recently, Inositol polyphosphate 4-phosphatase type II (INPP4B), a new factor in the regulation of the PI3K signaling module in tumors, was observed to preferentially dephosphorylate PI (3,4) P_2_ to produce PI (3) P and thereby block the activation of AKT [18, 19]. The suppressive function of INPP4B, akin to that of PTEN, was initially identified in breast cancer [20], and later confirmed in ovarian [21] and prostate cancers [22]. Interestingly, several recent reports have shown that INPP4B overexpression could be detected in other cancer contexts, such as PIK3CA-mutant breast cancer [23] and a subset of melanoma [24]. Notably, INPP4B was aberrantly overexpressed and emerged as an independent predictor of poor prognosis in AML patients with normal cytogenetics [25]. To date, however, the biological role of INPP4B in NPM1-mutated AML and the molecular mechanisms by which INPP4B contributes to leukemogenesis remain unclear.

In breast and other epithelial cancers, INPP4B has been predicted to be a tumor suppressor that blocks AKT activation. However, INPP4B expression is not associated with the changes in AKT phosphorylation status in leukemia, indicating that AKT-independent mechanisms are likely at play [25]. Serum and glucocorticoid-regulated kinase-3 (SGK3), another PI3K-dependent serine/threonine kinase, shares high structural and functional similarities with the AKT protein [26]. However, unlike AKT, SGK3 contains a unique N-terminal phox homology (PX) domain that binds to PI (3) P, thus targeting early endosomes where SGK3 is fully activated [27, 28]. Indeed, SGK3 emerges as an alternative downstream effector of INPP4B that diverges from canonical AKT signaling [29]. Recent studies have indicated an association between high INPP4B expression and SGK3 phosphorylation levels in PIK3CA-mutant breast cancers and melanoma, in which INPP4B-mediated activation of SGK3 enhances cell proliferation and promotes anchorage-independent cell growth [23, 24]. Herein, we report that INPP4B is frequently upregulated in NPM1-mutated AML, and promotes leukemia cell survival in a SGK3-dependent and AKT-independent manner. Increased INPP4B expression is partially caused by the NPM1 mutant through ERK/Ets-1 signaling. In addition, high INPP4B is associated with poor outcome in NPM1-mutated patients in our study. Previous reports and the present study suggest that INPP4B provides a survival advantage through the activation of SGK3 in NPM1-mutated leukemia cells. These findings further indicate that INPP4B might be a potential target for the treatment of NPM1-mutated AML.

## Methods

### The cancer genome atlas (TCGA) gene expression data analysis

Gene expression levels and clinical information of 200 AML patients were retrieved from The Cancer Genome Atlas (TCGA, http://www.cancergenome.nih.gov). A total of 171 samples had IlluminaGA RNA-Seq gene expression data. Clinical data and INPP4B mRNA expression data for AML samples were analyzed using the cBioPortal for Cancer Genomics. The *INPP4B* mRNA expression was compared between AML cases with the NPM1 mutation (*n* = 41) and those without the NPM1 mutation (*n* = 130).

### Patient samples

Peripheral blood samples of 36 AML patients, who had been recently diagnosed, including 22 NPM1-unmutated and 14 NPM1-mutated cases, were obtained from Southwest Hospital of the Third Military Medical University and the First Affiliated Hospital of Chongqing Medical University. Mononuclear cells were enriched by Ficoll gradient purification. The isolated mononuclear cells were used for analyses of NPM1-mA and INPP4B relative expression. Details of the Clinical characteristics of patients are provided in Table 1.

**Table 1:** Clinical Characteristics of Newly Diagnosed AML Patients

Characteristics	Median (range)	No. of cases
*Sex*		
Female		19
Male		17
Total		36
*Age*		
Median, years	53.8 (26–79)	
*WBC*		
Median, ×10^9^/L	44 (0.3–295.0)	
*Platelets*		
Median, ×10^9^/L	57.3 (3.0–655.0)	
*AML FAB subtype*		
AML without maturation: M1		4
AML with maturation: M2		6
Acute promyelocytic leukemia: M3		9
Acute myelomonocytic leukemia: M4		7
Acute monoblastic or monocytic leukemia: M5		9
Other subtype		1
*Karyotype*		
Normal		14
t (8;21)		5
t (15;17)		6
inv. (16)		7
Unknown		4
*Gene mutations*		
*NPM1*		14
*FLT3-ITD*		10
*WT1*		9
*CBFB-MYH11*		5

Abbreviations: *AML* acute myeloid leukemia, *WBC* white blood cell; FAB classification, French-American-British classification, a classification of acute leukemia produced by three-nation joint collaboration

### Cell cultures

Human myeloid leukemia cells HL60, KG1a, K562 and THP-1 were obtained from the American Type Culture Collection (ATCC, MD, USA). The OCI-AML3 AML cells harboring NPM1-mA [30] were obtained from Deutsche Sammlung von Mikroorganismen und Zellkulturen GmbH (DSMZ, Braunschweig, Germany). All cell lines were routinely cultured in RPMI 1640 medium (Gibco, MD, USA), supplemented with 10% fetal bovine serum (FBS; Gibco, MD, USA) and 1% penicillin and streptomycin (Beyotime, Shanghai, China) in a 5% CO_2_ humidified incubator at 37 °C.

### Reverse transcription PCR and quantitative real-time PCR

Total RNA was isolated using the TRIzol reagent (Takara, Kyoto, Japan), and transcribed into cDNA using the PrimeScript™ RT Reagent Kit (Takara, Kyoto, Japan). Quantitative real-time PCR (qRT-PCR) analysis was performed on an MJ Mini™ Gradient Thermal Cycler Real-Time PCR machine (Bio-Rad, CA, USA) with the SYBR Green reaction kit (KAPA Biosystems, MA, USA). The following primers were used for real-time amplification: *INPP4B* (Forward 5’-GGAAAGTGTGAGCGGAAAAG-3′ and Reverse 5′- CGAATTCGCATCCACTTATTG-3′); *NPM1-mA* (Forward F: 5′-TGGAGGTGGTAGCAAGGTTC-3′ and Reverse 5′-CTTCCTCC ACTGCCAGACAGA-3′); *SGK3* (Forward 5′-CTGAGATCTCACCATGCAAA GAGATCACACC-3′ and Reverse 5′-GGGGCTAGCTCACAAAAATAAG TCTTCT-3′); *Ets-1*(Forward 5′-GTCGTGGTAAACTCGG-3′ and Reverse 5′-CAG CAGGAATGACAGG-3′); *β-actin* (Forward 5′-TAGTTGCGTTACACCCTTTC TTG-3′ and Reverse 5′-TGCTGTCACCTTCA CCGTTC-3′). The mRNA expression levels were analyzed using the 2^- ΔΔCt^ method and expressed as a fold change.

### Western blotting

The cultured cells were washed and lysed in cell extraction buffer. Equal amounts of extracts were loaded into sodium dodecyl sulfate (SDS) polyacrylamide gels for electrophoresis and transferred onto polyvinylidene difluoride (PVDF) membranes. The membranes were blocked in 5% low-fat dry milk for 3 h, and then incubated overnight at 4 °C with primary antibodies against INPP4B, p-SGK3^T320^, SGK3, p-AKT^T308^, AKT, p-ERK, ERK (Cell Signaling Technology, MA, USA); p-Ets-1, Ets-1, Flag (Bioworld Technology Inc. MN, USA); NPM1-mA (Abcam, Cambrige, UK) and β-actin (Santa Cruz Biotechnology Inc. CA, USA) as loading control. Membranes were washed in Tris-buffered saline (TBS) (10 mM Tris-HCl pH 8, 150 mM NaCl) containing 0.1% Tween 20, and then incubated with HRP-conjugated secondary antibody for 1 h, and subsequently exposed to enhanced chemiluminescence substrate (Millipore, MA, USA). Membrane blot signals were detected using the Bio-Rad Gel Imaging System on cool image workstation II (Viagene, FL, USA). Quantification of protein expression was normalized against the β-actin protein expression using imaging software.

### Delivery of siRNA and cell transfection

The siRNA targeting INPP4B, SGK3, Ets-1 and control siRNA were purchased from Genechem (Shanghai, China). The OCI-AML3 cells were transfected with siRNA using the Rfect^PM^ siRNA Transfection Reagent (BaiDai, Changzhou, China) according to the manufacturer’s instructions. After 48 h of transfection, the cells were collected for qRT-PCR or western blotting analysis. The sequences of siRNA were as follows: siINPP4B1 (sense: 5’-CCAGGAGGCAUUCUUAAGATT-3′; antisense: 5’-UCUUAAGAAUGCCUCCUGGTT-3′); siINPP4B2 (sense: 5’-GCCGCAAACUGAAUGGUAUTT-3′; antisense: 5’-AUACCAUUCAGUUUGCGGCTT-3′); siSGK3 (sense: 5’-GCAGGACUAAACGAAUUCATT-3′; antisense: 5’-UGAAUUCGUUUA GUCCUGCTT-3′); siEts-1 (sense: 5’-ACUUGCUACCAUCCCGUAC-3′; antisense: 5’-GUACGGGAUGGUAGCAAGU-3′); Control (sense: 5’-UUCUUCGAACGUGUCACGUTT-3′; antisense: 5’-ACGUGACACGUUCGGAGAATT-3′).

### Lentiviral vectors and cell infection

The lentivirus-based short hairpin RNA (shRNA) vectors targeting *INPP4B* (5’-CCATCTGAGTATCCCATCTAT-3′) and scramble lentiviral vectors were purchased from Genechem (Shanghai, China). The OCI-AML3 cells were infected with lentivirus for 48 h in the presence of 5 μg/mL polybrene (Sigma, CA, USA), after which they were subjected to 2 μg/mL puromycin selection for 7 d (Sigma, CA, USA). The puromycin-resistant cells were isolated and propagated for further analysis.

### Plasmids and cell transfection

The pEAK-Flag/INPP4B and pCMV-Flag/SGK3 plasmids were purchased from Addgene (http://www.addgene.org). The pEGFPC1-NPM1-mA, pEGFPC1-NPM1-wt and empty pEGFPC1 were kindly provided by Dr. Falini B (Institute of Hematology, University of Perugia, Perugia, Italy). All transfection experiments were performed using the xfect™ reagent (Clontech, CA, USA) according to the manufacturer’s instructions. After 48 h of transfection, the cells were collected for qRT-PCR or western blotting analysis.

### Cell viability assay

Cell viability was determined by the Cell Counting Kit-8 (CCK8, Dojindo Laboratories, Japan), according to the manufacturer’s instructions. Cells were seeded into 96-well plates (Corning, NY, USA) in triplicate at a density of 5 × 10^3^ cells per well with RPMI-1640 containing 10% FBS. The cell numbers were quantified at the indicated time points with the CCK8 (10 μl/well at 37 °C for 2 h), and the numbers of cells per well were determined by measuring absorbance at 450 nm using the microplate reader (Eon, BioTeck, CA, USA). The cell growth curves were plotted with the cell number values as the ordinate and time as the abscissa. Each experiment was performed in triplicate.

### Colony formation assay

A methylcellulose clonogenic assay was carried out to determine cell colony forming ability, by planting 1 × 10^3^ cells per well in triplicate in a 24 well-plate, and maintaining those cells in RPMI 1640 medium containing 20% FBS at 37 °C in an incubator. Colony numbers were scored 10 d later. The colony forming units were counted using an inverted microscope.

### Inhibitor treatment

The PI3K inhibitor, LY294002 (30 μM); the AKT selective inhibitor, MK-2206 (5 μM); and the mTOR inhibitor, rapamycin (5 μM) were used to treat OCI-AML3 cells for 24 h and the treated cells were harvested for western blotting. The ERK inhibitor PD98059 was used to treat OCI-AML3 cells with different concentrations (0, 10, 20 and 40 μM) for 24 h and the treated cells were harvested for qRT-PCR and western blotting. These inhibitors were purchased from Selleck Chemicals (Selleckchem, TX, USA).

### PI (3,4) P_2_ and PI (3) P enzyme-linked immunosorbent assay (ELISA)

Cellular PI (3,4) P_2_ and PI (3) P were quantitated using RY-02853 Human PI (3,4) P_2_ ELISA kit and RY-02851 Human PI (3) P ELISA kit, respectively, obtained from Runyu Biotechnology (Shanghai, China), according to the manufacturer’s instruction. The results were recorded and analyzed using the microplate reader (Eon, BioTeck, CA, USA).

### Survival analysis

Gene expression levels and clinical survival information of 153 AML patients, including 38 patients harboring NPM1 mutations, were retrieved from TCGA dataset. All patients were stratified by INPP4B expression levels into quartiles, to categorize patients into either a high cohort or low cohort. Kaplan-Meier data of AML patients and NPM1-mutated patients were used to analyze the overall survival (OS) and the three-year event free survival (EFS). Details of the clinical characteristics according to high or low INPP4B expression among NPM1-mutated patients from TCGA dataset are provided in Table 2.

**Table 2:** Clinical Characteristics of NPM1-mutated AML Patients with Low or High INPP4B Expression

Characteristics	Low INPP4B (*n* = 29)	High INPP4B (*n* = 9)
*Sex*		
Female	15	6
Male	14	3
*Age, years*		
Median, range	52.93 (21–81)	50.56 (21–82)
*WBC, ×10* ^*9*^ */L*		
Median, range	61.58 (5–137)	49.78 (1–134)
*Platelets, ×10* ^*9*^ */L*		
Median, range	53.86 (8–174)	63.56 (11–232)
*Bone marrow blast, %*		
Median, range	78.93 (48–98)	70.11 (41–95)
*FAB classification*		
M0	0	1
M1	11	2
M2	3	4
M3	0	0
M4	10	0
M5	5	2
M6	0	0
M7	0	0
*Cytogenetics*		
Normal	27	7
Abnormal	0	1
Unknown	2	1
*Cytogenetic risk*		
Favorable	0	0
Intermediate	28	7
Adverse	0	1
Unknown	1	1
*Gene mutations*		
*FLT3-ITD*	16	6
*IDH1*	8	0

Abbreviations: *AML* acute myeloid leukemia, *WBC* white blood cell, *FAB* classification, French-American-British classification, a classification of acute leukemia produced by three-nation joint collaboration

### Statistical analysis

All data were derived from three independent experiments and the results were summarized and represented as mean ± s.d. Statistical analysis was performed, using the SPSS (Version 17.0) and GraphPad (Prism 5.0) software programs. The statistical significance of differences between each group was analyzed using the unpaired Students’ *t*-test. The Kaplan–Meier survival data were analyzed using the long-rank test. Any *p*-value <0.05 was considered statistically significant.

## Results

### INPP4B is highly expressed in leukemia with the NPM1 mutation

**Fig. 1:**
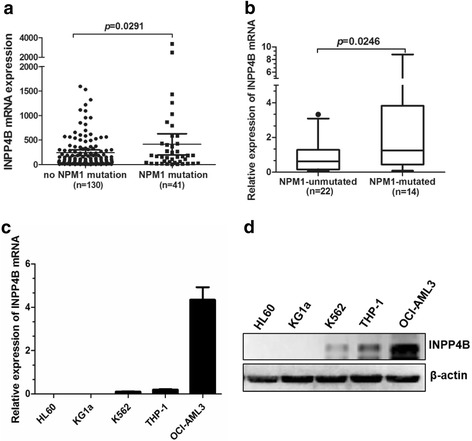
High expression levels of INPP4B in leukemia cells with the NPM1 mutation. **a** RNA-seq mRNA expression data from the TCGA database were used to compare *INPP4B* expression between AML patients with (*n* = 41) and without NPM1 mutation (*n* = 130). * *p* < 0.05. **b** The mRNA levels of *INPP4B* in primary NPM1-mutated AML cases (*n* = 14) were assessed by qRT-PCR, and compared to NPM1-unmutated AML cases (*n* = 22). **c** qRT-PCR analysis of *INPP4B* mRNA expression from the indicated myeloid leukemia cell lines. **d** Western blotting analysis of INPP4B and β-actin (as a loading control). Data were represented as mean ± s.d. of three individual experiments

To determine the expression levels of INPP4B in AML with the NPM1 mutation, we first analyzed the publicly available TCGA RNA-seq dataset of 171 AML patients. The results showed that the *INPP4B* transcript levels were increased among the cases of AML with the NPM1 mutation, as compared to those without the NPM1 mutation (*p* = 0.0291, Fig. 1a). Consistent with these findings, we examined the mRNA levels of *INPP4B* by qRT-PCR from 36 primary AML blasts and found that INPP4B was more highly expressed in NPM1-mutated AML (*n* = 14), as compared to NPM1-unmutated AML (*n* = 22) cases (*p* = 0.0246, Fig. 1b). To confirm our theory, we then assessed *INPP4B* mRNA and protein abundance across a panel of human myeloid leukemia cell lines. The *INPP4B* mRNA levels were readily detected in all but two cell lines (HL60 and KG1a) at various levels. Interestingly, the OCI-AML3 cells that naturally carried NPM1-mA, showed increased *INPP4B* mRNA expression (Fig. 1c). Similarly, INPP4B protein levels were also increased in OCI-AML3 cells (Fig. 1d). These results demonstrate that INPP4B showed relatively high expression in NPM1-mutated AML.

### INPP4B promotes cell proliferation in OCI-AML3 cells

**Fig. 2:**
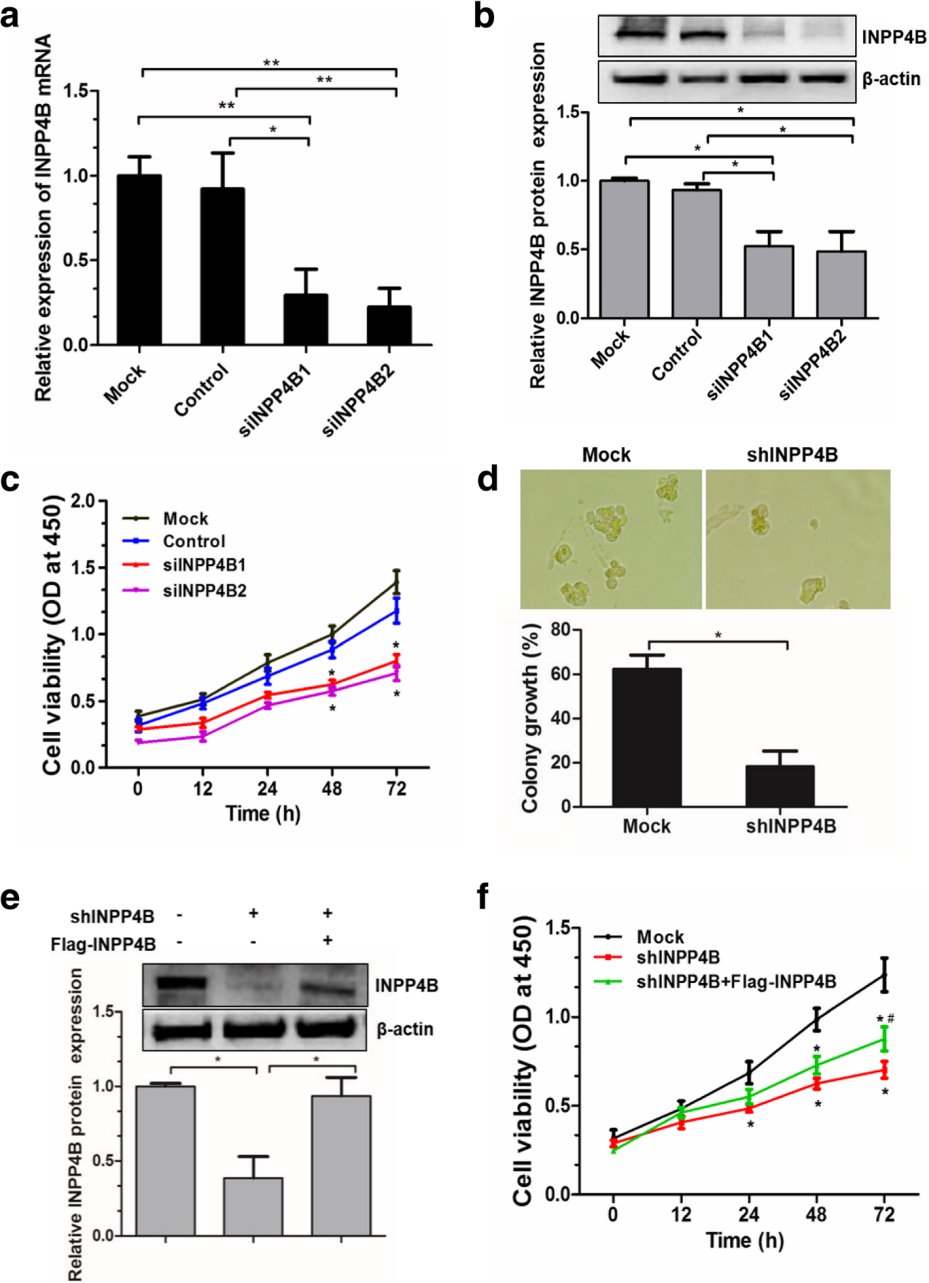
INPP4B promotes cell proliferation in OCI-AML3 cells. (**a**) qRT-PCR and (**b**) western blotting analysis of INPP4B expression from the OCI-AML3 cells transduced with the control siRNA or siINPP4B. **c** CCK-8 assay analysis of cell proliferation activity in the siINPP4B transduced cells. **d** The OCI-AML3 cells stably infected with shRNA lentivirus targeting INPP4B were subjected to colony forming assays. **e** The INPP4B-silenced OCI-AML3 cells were transfected with the pEAK-Flag/INPP4B plasmids, western blotting analysis of INPP4B protein levels and quantified using image software normalized against β-actin. **f** CCK-8 assay analysis of cell proliferation in INPP4B-silenced OCI-AML3 cells, followed by Flag-INPP4B introduction. Data were represented as mean ± s.d. of three individual experiments. * *p* < 0.05, ** *p* < 0.01, as comparison to mock group; # *p* < 0.05, as comparison to shINPP4B group

To evaluate the functional significance of INPP4B upregulation on cell proliferation in NPM1-mutated leukemia, OCI-AML3 cells were transfected with two individual siRNAs to silence INPP4B. Once the decreased levels of siRNA-mediated *INPP4B* mRNA and protein were successfully confirmed in OCI-AML3 cells (Fig. 2a and b), we found that the loss of INPP4B significantly inhibited cell proliferation in vitro (Fig. 2c). In addition, INPP4B silencing markedly reduced the number of colonies, as evidenced by a significant reduction in colony forming potential, in comparison to the controls (Fig. 2d).

We further verified the effects of INPP4B on OCI-AML3 cell proliferation through a rescue experiment. First, a lentiviral vector was used to establish a stable cell line with shRNA-mediated knockdown of *INPP4B*, and INPP4B protein was successfully deleted in OCI-AML3 cells (Fig. 2e). The pEAK-Flag/INPP4B plasmids were then introduced into *INPP4B*-silenced OCI-AML3 cells and the INPP4B protein was successfully recovered (Fig. 2e). Notably, forced expression of INPP4B could reverse the inhibitory effects of *INPP4B* knockdown on cell proliferation (Fig. 2f). Taken together, our data indicate that INPP4B was, at least in part, required for cell proliferation in OCI-AML3 leukemia cells.

### INPP4B mediates SGK3 activation in OCI-AML3 cells

**Fig. 3:**
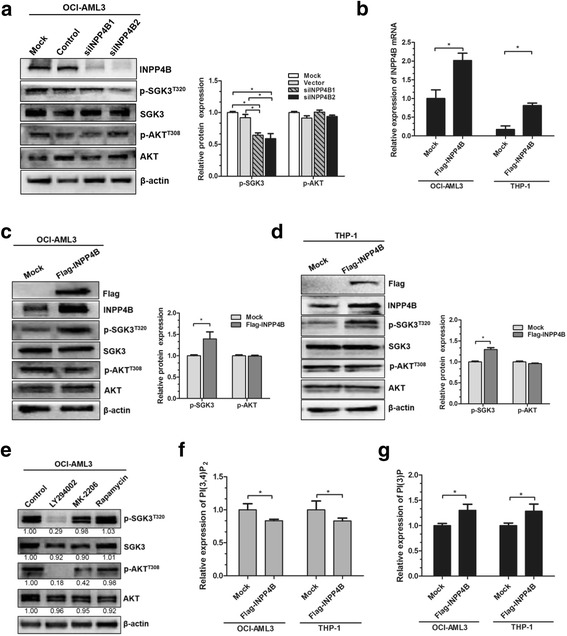
INPP4B mediates SGK3 activation in OCI-AML3 cells. **a** Western blotting analysis of INPP4B, p-SGK3^T320^, SGK3, p-AKT^T308^ and AKT from OCI-AML3 cells transduced with the control siRNA or siINPP4B. **b** qRT-PCR analysis of INPP4B mRNA expression from OCI-AML3 and THP-1 cells transduced with the pEAK-Flag/INPP4B plasmids. **c-d** Western blotting analysis of Flag, INPP4B, p-SGK3^T320^, SGK3, p-AKT^T308^ and AKT from the INPP4B transduced cells. **e** Western blotting analysis of p-SGK3^T320^, SGK3, p-AKT^T308^ and AKT from the OCI-AML3 cells treated with LY294002 (30 μM), MK-2206 (5 μM) and Rapamycin (5 μM), respectively. Proteins were quantified using image software and normalized against β-actin. The relative abundance of (**f**) PI (3,4) P_2_ and (**g**) PI (3) P in the INPP4B transduced cells was measured using ELISA. Data were represented as mean ± s.d. of three individual experiments. * *p* < 0.05

To explore the potential mechanisms by which INPP4B contributes to cell proliferation in NPM1-mutated leukemia, we performed siRNA-mediated knockdown of *INPP4B* in OCI-AML3 cells, and the phosphorylation (activation) of SGK3 and AKT was then monitored. Our results revealed that INPP4B depletion caused a significant reduction in SGK3 phosphorylation levels, but did not affect AKT activation in OCI-AML3 cells (Fig. 3a). To confirm this notion even further, the pEAK-Flag/INPP4B plasmids were transfected into two cell lines and *INPP4B* mRNA levels were increased **(**Fig. 3b**)**. As expected, INPP4B overexpression led to significantly elevated p-SGK3^T320^ levels in OCI-AML3 cells, but did not affect p-AKT^T308^ levels (Fig. 3c). Similar results were observed in THP-1 leukemia cells (Fig. 3d). Furthermore, the effects of the PI3K signaling pathway inhibitors on the levels of p-SGK3 in OCI-AML3 cells were tested. As shown in Fig. 3e, treatment with the PI3K inhibitor, LY294002, evidently reduced p-SGK3 levels; however, no obvious reduction in p-SGK3 levels was observed in the groups treated with the AKT-selective inhibitor, MK-2206 and the mTOR inhibitor, rapamycin. Previous studies verified that the activation of SGK3 would occur at sites of PI (3) P accumulation [31]. We thus tested the impact of INPP4B on cellular levels of PI (3,4) P_2_ and PI (3) P. The results showed that introduction of exogenous INPP4B decreased PI (3,4) P_2_ levels and increased PI (3) P levels in leukemia cells **(**Fig. 3f, g**)**. Collectively, our results indicate that INPP4B mediated activation of the PI3K downstream factor, SGK3, but not AKT, in OCI-AML3 cells.

### SGK3 is required for INPP4B-induced cell proliferation in OCI-AML3 cells

**Fig. 4:**
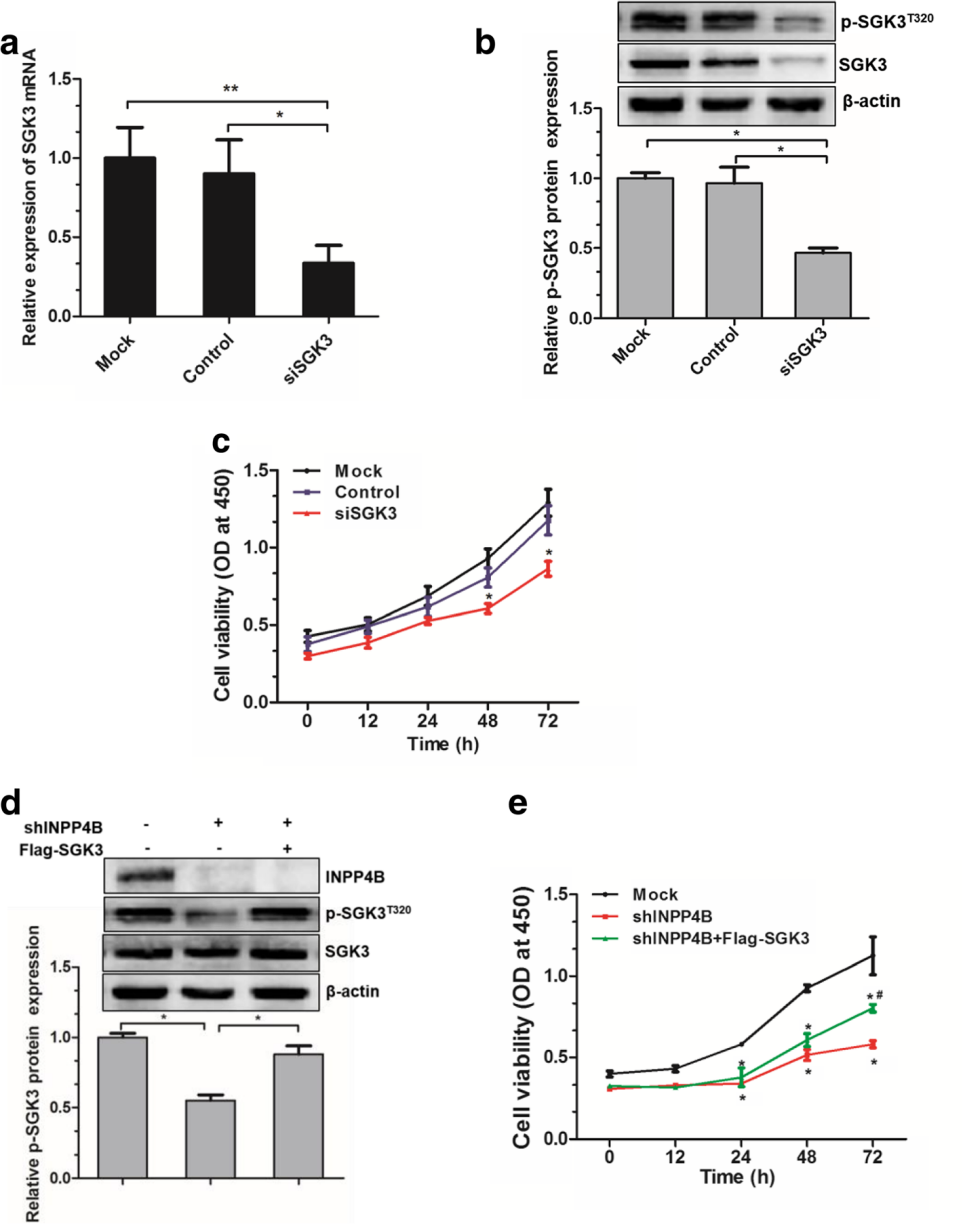
SGK3 is required for INPP4B-induced cell proliferation in OCI-AML3 cells. qRT-PCR (**a**) and western blotting (**b**) analysis of SGK3 expression from the OCI-AML3 cells transduced with the control siRNA or siSGK3. **c** CCK-8 assay analysis of cell proliferation activity in the siSGK3 transduced cells. **d** The INPP4B-silenced OCI-AML3 cells were transfected with the pCMV-Flag/SGK3 plasmids, western blotting analysis of SGK3, p-SGK3^T320^ and INPP4B. Proteins were quantified using image software and normalized against β-actin. **e** CCK-8 assay analysis of cell proliferation in INPP4B-silenced OCI-AML3 cells, followed by Flag-SGK3 introduction. Data were represented as mean ± s.d. of three individual experiments. * *p* < 0.05, as comparison to mock or control group, respectively; # *p* < 0.05, as comparison to shINPP4B group

Based on the foregoing data, we hypothesized that SGK3 might provide an advantage for INPP4B-induced cell proliferation in NPM1-mutated leukemia. To test this, we transfected the siRNA targeting SGK3 into OCI-AML3 cells and found that *SGK3* mRNA and protein levels were indeed reduced (Fig. 4a and b). Notably, SGK3 depletion markedly repressed cell proliferation in OCI-AML3 cells (Fig. 4c). A rescue assay was then used to verify the effects of SGK3 on INPP4B-induced cell proliferation. The results showed that reduced SGK3 phosphorylation induced by INPP4B silencing was rescued by the introduction of exogenous SGK3 (Fig. 4d). Furthermore, SGK3 overexpression partially neutralized the inhibitory effect of shINPP4B-induced cell proliferation (Fig. 4e). These results suggest that SGK3 was required for INPP4B-induced cell proliferation in OCI-AML3 cells.

### INPP4B is upregulated by NPM1-mA via ERK/Ets-1 signaling in leukemia cells

**Fig. 5:**
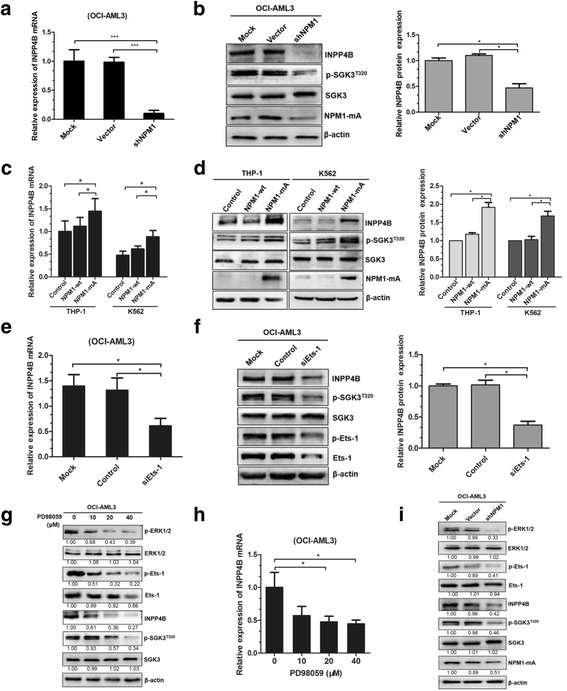
INPP4B is upregulated by NPM1-mA in leukemia cells via ERK/Ets-1 signaling. (**a**) qRT-PCR analysis of *INPP4B* mRNA expression, (**b**) western blotting analysis of INPP4B, p-SGK3^T320^, SGK3 and NPM1-mA from the NPM1-mA-silenced OCI-AML3 cells. (**c**) qRT-PCR analysis of *INPP4B* mRNA expression, (**d**) western blotting analysis of INPP4B, p-SGK3^T320^, SGK3 and NPM1-mA from the THP-1 and K562 cells transduced with the plasmids expressing NPM1-wt or NPM1-mA. (**e**) qRT-PCR analysis of *INPP4B* mRNA expression, (**f**) western blotting analysis of INPP4B, p-SGK3^T320^, SGK3, p-Ets-1 and Ets-1 from the OCI-AML3 cells transfected with the control siRNA or siEts-1. (**g**) Western blotting analysis of p-ERK, ERK, p-Ets-1, Ets-1, INPP4B, p-SGK3^T320^ and SGK3, (**h**) qRT-PCR analysis of *INPP4B* mRNA expression from the OCI-AML3 cells treated with different concentration of PD98059 (0, 10, 20 and 40 μM). **i** Western blotting analysis of p-ERK, ERK, p-Ets-1, Ets-1, INPP4B, p-SGK3^T320^, SGK3 and NPM1-mA from the NPM1-mA-silenced OCI-AML3 cells. Proteins were quantified using image software and normalized against β-actin. Data were represented as mean ± s.d. of three individual experiments. * *p* < 0.05, *** *p* < 0.001

We evaluated the potential molecular mechanism underlying INPP4B upregulation in leukemia cells with NPM1-mA. First, stably NPM1-silenced OCI-AML3 cells, for which treatment with shRNA targeting *NPM1* depleted both NPM1-wt and NPM1-mA levels, were established according to previously described protocols [32] and used for further analysis. We found that the loss of NPM1-mA significantly reduced *INPP4B* mRNA levels (Fig. 5a). Moreover, INPP4B protein levels and downstream phosphorylated SGK3 were considerably reduced in the NPM1-mA silenced group (Fig. 5b). This hypothesis was further confirmed, as we found that *INPP4B* mRNA levels were increased upon ectopic overexpression of NPM1-mA in THP-1 and K562 leukemia cell lines (Fig. 5c). Moreover, INPP4B and phosphorylated SGK3 proteins were consistently elevated, due to the introduction of NPM1-mA (Fig. 5d). This suggests that upregulation of INPP4B/SGK3 was at least partially caused by NPM1-mA expression in leukemia cells.

A recent study demonstrated that the increase in INPP4B is due to Ets-1-mediated transcriptional upregulation in colon cancer cells [33]. To test this effect in NPM1-mutated AML, the siRNA targeting Ets-1 was transfected into OCI-AML3 cells, and *Ets-1* knockdown was found to reduce *INPP4B* mRNA expression significantly (Fig. 5e). In addition, deletion of *Ets-1* evidently downregulated INPP4B protein levels (Fig. 5f). Given that Ets-1 could be phosphorylated by ERK signaling, and thereby enhanced its transactivation [34], we thus treated the OCI-AML3 cells with the ERK inhibitor (PD98059) and observed that the levels of p-Ets-1 were decreased in a dose-dependent manner (Fig. 5g). Meanwhile, the INPP4B protein and mRNA levels were considerably reduced (Fig. 5g and h). We previously determined that the ERK activation was continuously increased by NPM1-mA in leukemia cells [35]. Next, we observed that NPM1-mA knockdown impaired ERK/Ets-1 signaling and further reduced INPP4B protein levels in OCI-AML3 cells (Fig. 5i). Taken together, these findings indicate that INPP4B expression might be upregulated by NPM1-mA via ERK/Ets-1 signaling in leukemia cells.

### NPM1-mA-mediated INPP4B upregulation promotes cell proliferation in OCI-AML3 cells

**Fig. 6:**
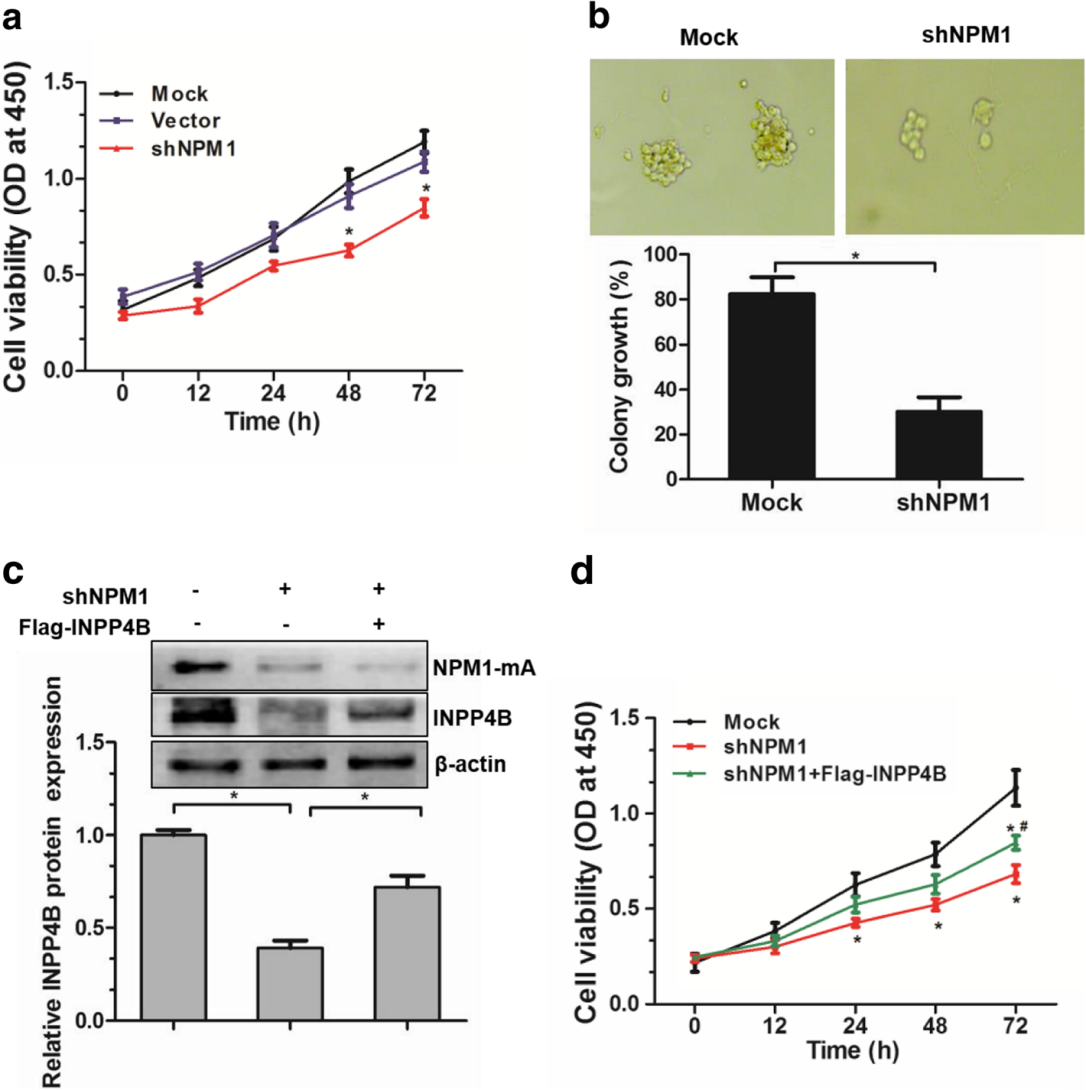
NPM1-mA-mediated INPP4B upregulation promotes cell proliferation in OCI-AML3 cells. The NPM1-mA-silenced OCI-AML3 cells were subjected to (**a**) CCK8 assays and (**b**) colony forming assays. **c** The NPM1-mA-silenced OCI-AML3 cells were transfected with the pEAK-Flag/INPP4B plasmids, western blotting analysis of INPP4B and NPM1-mA. Proteins were quantified using image software and normalized against β-actin. **d** CCK-8 assay analysis of cell proliferation in NPM1-mA-silenced OCI-AML3 cells, followed by Flag-INPP4B introduction. Data were represented as mean ± s.d. of three individual experiments. * *p* < 0.05, as comparison to mock or vector group, respectively; # *p* < 0.05, as comparison to the shNPM1 group

Given that NPM1-mA enhanced INPP4B expression, we then validated the impact of NPM1-mA on the proliferation of leukemia cells. The results showed that knockdown of NPM1-mA significantly attenuated cell proliferation in OCI-AML3 cells, as compared to the control group (Fig. 6a). In addition, the colony formation assay showed a decreased colony size and a lower proportion of colony forming units in the NPM1-mA-silenced OCI-AML3 cells (Fig. 6b). To further evaluate the pivotal role of NPM1-mA mediated INPP4B upregulation in cell growth, we analyzed cell proliferation, using a rescue assay in NPM1-mA silenced cells. The results showed that INPP4B protein levels were successfully recovered upon INPP4B overexpression (Fig. 6c). Importantly, the inhibiting effect of NPM1-mA depletion-mediated cell proliferation was reversed by re-expression of INPP4B (Fig. 6d). These results indicate that INPP4B upregulation mediated by NPM1-mA promotes cell proliferation in OCI-AML3 cells.

### High expression of INPP4B is associated with poor survival outcome in NPM1-mutated leukemia

**Fig. 7:**
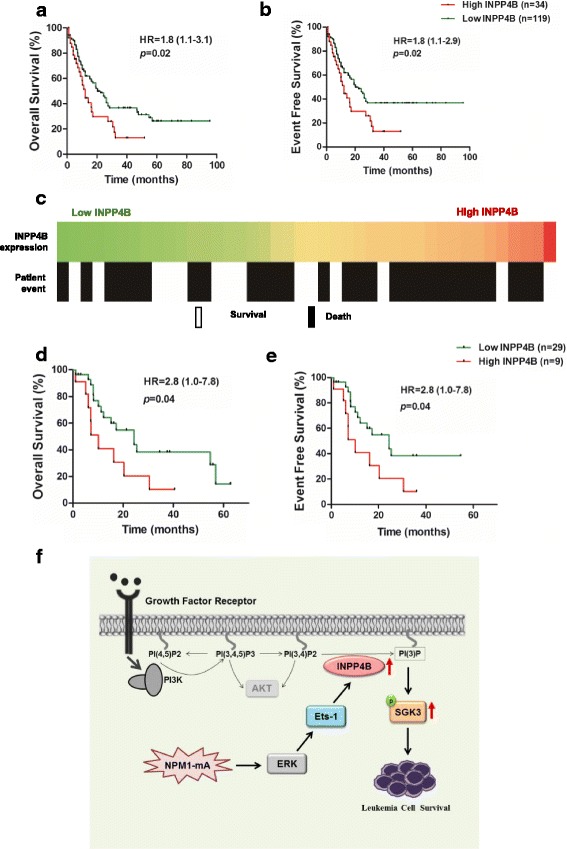
High expression of INPP4B is associated with poor survival outcome in NPM1-mutated leukemia. Kaplan-Meier survival data of 153 AML patients were used to analysis (**a**) OS and (**b**) EFS curves according to INPP4B levels. **c** Heatmap of 38 primary AML samples with NPM1 mutation from TCGA dataset in which INPP4B expression was aligned with patient event (Survival or Death). Kaplan-Meier survival data of patients with NPM1-mutated AML were used to analysis (**d**) OS and (**e**) EFS curves according to INPP4B levels. **f** Schematic diagram describing the functional significance of INPP4B in the NPM1-mutated leukemia cells. INPP4B promotes leukemia cell survival in a SGK3-dependent and AKT-independent manner. The expression of INPP4B partially upregulated by NPM1-mA is due to ERK/Ets-1 signaling

To investigate the function of INPP4B in AML patients with the NPM1 mutation, we investigated the prognostic value of INPP4B using the dataset of TCGA, for which clinical data were available. First, Kaplan–Meier survival analysis was performed on 153 AML samples. The results showed that high INPP4B cohort had significant shorter OS (median, 14.3 vs 22.5 months; *p* = 0.02, Fig. 7a) and the three-year EFS (median, 14.3 vs 22.5 months; *p* = 0.02, Fig. 7b), compared with low INPP4B cohort of AML patients. Noticeably, we observed that NPM1-mutated patients with high INPP4B expression showed an approximate correlation with inferior survival (Fig. 7c). Furthermore, Kaplan-Meier survival analysis of 38 NPM1-mutated samples revealed that the OS (median, 10.1 vs 24.3 months; *p* = 0.04, Fig. 7d) and the three-year EFS (median, 10.1 vs 24.3 months; *p* = 0.04, Fig. 7e) was evidently shorter in high INPP4B cohort, as compared to the low INPP4B cohort. Moreover, the samples from TCGA confirmed the poor outcome (hazard ratio [HR] = 2.8) of high INPP4B expression in NPM1-mutated AML. These clinical data from TCGA dataset indicate that high expression of INPP4B showed a trend towards poor prognosis in AML cases with NPM1 mutations.

## Discussion

The *NPM1* mutations are among the most frequent genetic alterations in AML, especially in cases with a normal karyotype. However, the pathogenesis of NPM1-mutated AML has not been fully elucidated. Herein, our data demonstrate that INPP4B functions to promote leukemia cell survival in a SGK3-dependent manner, high levels of INPP4B are at least partially caused by the NPM1 mutant via ERK/Ets-1 signaling, and high INPP4B is potentially correlated with poor clinical outcome in NPM1-mutated leukemia (Fig. 7f).

It has been known that INPP4B is a phosphoinositide phosphatase with pleiotropic functions in various cellular processes [36]. As a novel factor in the PI3K signaling pathway, INPP4B has been found to play a tumor suppressive role in prostate, breast, and ovarian cancers, and possibly in leukemia [37, 38]. However, unexpected findings from recent reports indicate that INPP4B function might be more complicated than previously thought [39]. In the present study, we analyzed *INPP4B* mRNA levels in NPM1-mutated AML patients from the dataset of TCGA and further detected INPP4B expression in NPM1-mutated AML primary blasts and NPM1-mA positive OCI-AML3 cells. These results revealed relative overexpression of INPP4B in NPM1-mutated AML. The overexpression of INPP4B first became evident after the analysis of gene expression in leukemic blasts from BCR/ABL-positive pediatric acute lymphoblastic leukemia [40]. High INPP4B expression was recently reported in a subset of AML patients with lower response rates to chemotherapy and shorter survival [41]. Next, we explored the biological effects of INPP4B overexpression in NPM1-mutated leukemia. We observed that knockdown of *INPP4B* resulted in inhibition of OCI-AML3 cell proliferation in vitro, and conversely, recovered *INPP4B* could rescue this inhibitory effect*,* which confirms the gain-of-function of *INPP4B* in NPM1-mutated AML. Our findings were consistent with those of a previous study, in which reported that the introduction of *INPP4B* conferred a significant increase in the colony forming potential in OCI-AML3 cells [41]. Moreover, Rijal et al. [25] reported that ectopic overexpression of *INPP4B* enhanced leukemic resistance to cytosine arabinoside (Ara-C). These findings and our data imply that the tumor suppressor gene *INPP4B* plays a potential oncogenic role in NPM1-mutated leukemia. In a future study, the function of INPP4B in mouse knock-in models that mimic human NPM1-mutated AML are worthy to be further investigated.

The INPP4B protein is a phosphoinositide phosphatase and acts as an important regulator in PI3K pathway-associated cancer [42]. In the present study, we found that introduction of INPP4B elevated SGK3 phosphorylation and knockdown of INPP4B reduced SGK3 phosphorylation in OCI-AML3 cells. These results are consistent with those of another report, in which the expression of INPP4B leads to enhanced SGK3 activation in PIK3CA-mutated breast cancer cells [23]. Because AKT is the canonical downstream effector of INPP4B in the PI3K pathway and INPP4B has the seemingly paradoxical role in AKT activation in a variety of different types of cancers [42], we tested whether INPP4B was involved in the regulation of phosphorylated AKT in leukemia cells. Surprisingly, our data demonstrate that loss or gain of INPP4B did not appear to affect AKT activation in NPM1-mutated leukemia cells. These results are consistent with those of a previous report of Rijal et al. [25], who reported no correlation between endogenous INPP4B protein levels and AKT phosphorylation status in AML. Next, we treated OCI-AML3 cells with PI3K pathway inhibitors and found that the PI3K inhibitor, LY294002 (but not the AKT-selective inhibitor, MK-2206 and the mTOR inhibitor, rapamycin) markedly reduced the p-SGK3 levels. These findings and our data indicate that INPP4B mediates activation of the PI3K downstream factor, SGK3, but not AKT in NPM1-mutated leukemia. It is well known that INPP4B preferentially hydrolyzes PI (3,4) P_2_ to produce PI (3) P through its lipid phosphatase activity [39]. Importantly, SGK3 phosphorylation and subsequent activation are dependent on binding to the PI (3) P [43]. In our study, we observed that introduction of exogenous INPP4B increased PI (3) P levels in OCI-AML3 cell. These results reveal that enhanced activation of SGK3 mediated by INPP4B might be due to the accumulation of PI (3) P in NPM1-mutated AML. Of note, INPP4B possesses both lipid and protein phosphatase activity [44], which remains possible that INPP4B also regulates phosphorylated SGK3 status through its protein phosphatase activity. Recently, Lopez et al. [44] has reported that K846M INPP4B mutant lacks lipid phosphatase activity but retains protein phosphatase activity. In the future study, we will further investigate the effects of INPP4B phosphatase activity on SGK3 using the K846M INPP4B mutant.

Considering the activation of SGK3 in NPM1-mutated leukemia cells, we assessed the role of SGK3 in INPP4B-mediated cell proliferation. The results from our experiments revealed that depletion of SGK3 led to significant inhibition of cell proliferation. We also performed a rescue assay and found that ectopic expression of SGK3 could reverse shINPP4B-induced inhibition of cell proliferation. Collectively, these findings support the hypothesis that INPP4B activates SGK3 signaling, to promote cell proliferation in NPM1-mutated leukemia. Several studies have demonstrated that SGK3 contributes to INPP4B-mediated cell proliferation in colon cancer [33] and a subset of melanomas cells [24]. In addition, INPP4B has been known to activate SGK3 and drive tumorigenesis in a subset of breast cancers with low levels of AKT [23]. Recently, a study has reported that INPP4B dephosphorylates tumor suppressor PTEN through its protein phosphatase activity and subsequent degradation of PTEN, thereby promotes cell proliferation of colon cancer [33]. Thus, in addition to SGK3, the other potential mechanisms underlying oncogenic role of INPP4B in NPM1-mutated leukemia cells needs to be determined.

Because INPP4B is aberrantly expressed in NPM1-mutated AML, the question is whether high expression of INPP4B in leukemic cells correlates with NPM1 mutations. We investigated the effects of NPM1 mutation on INPP4B expression. The results revealed that enforced expression of NPM1-mA increased *INPP4B* mRNA and protein levels, whereas knockdown of NPM1-mA had the opposite effect. As INPP4B protein expression seems largely correlated with its mRNA expression in NPM1-mutated leukemia cells (Fig. 5a-d), it is likely that INPP4B is elevated by transcriptional mechanisms. We next searched for the specific transcription factors involved in this process. A recent paper has identified that the increased expression of INPP4B is due to transcriptional upregulation mediated by the transcription factor Ets-1 in colon cancer cells [33]. In the present study, we found that siRNA-mediated knockdown of Ets-1 significantly downregulated *INPP4B* mRNA and protein levels in OCI-AML3 cells. It has been well documented that ERK activation is specifically required for the transcriptional function of Ets-1 [45]. Moreover, our previous study has verified that ERK signaling is continuously activated by NPM1-mA [35]. In this study, we treated OCI-AML3 cells with the ERK inhibitor, PD98059, and found that this treatment inhibited Ets-1 phosphorylation and further reduced the INPP4B levels in a dose-dependent manner. More notably, the loss of NPM1-mA weakened ERK/Ets-1 signaling and then reduced INPP4B protein levels. These results indicate that INPP4B is partially upregulated by NPM1-mA via ERK/Ets1 signaling. Previous studies have reported that AML cases carrying NPM1 mutations are generally associated with other common mutations (FLT3-ITD or DNMT3A) [46, 47]. Recently, a study from TCGA Research Network showed the complex relationships of cooperation or mutual exclusivity among these mutations related to AML [48]. Future studies will determine whether there is any association between the presence of other mutations and the level of INPP4B in NPM1-mutated patients. In addition, NPM1 is a nucleolar phosphoprotein and its phosphorylation status alters its functions. Previous study has shown that NPM1 could be dephosphorylated on Thr199 by the Ser/Thr protein phosphatase PP1β in response to DNA damage [49]. Considering the fact that INPP4B has protein tyrosine phosphatase activity [44] and Ser/Thr phosphatase activity [33], whether NPM1 might be a substrate of the INPP4B protein in leukemia remains to be clarified.

Next, we also observed that knockdown of NPM1-mA significantly inhibited cell proliferation in OCI-AML3 cells and the introduction of INPP4B successfully reversed this inhibitory effect. These observations indicate that NPM1-mA enhanced INPP4B expression and promoted cell survival in AML. We have previously identified the crucial role of NPM1 mutations in the invasion phenotype [35], cell differentiation block [32], and autophagic activity [50] in AML. Finally, we evaluated the clinical significance of INPP4B in NPM1-mutated AML cases derived from the TCGA dataset. High levels of INPP4B delineated a poorer prognosis in AML patients. Importantly, NPM1-mutated patients with high INPP4B tended to have shorter survival outcome. Our results support this observation, by showing that high INPP4B levels are associated with a poor outcome in AML among six independent gene expression datasets [41].

## Conclusions

In summary, for the first time to our knowledge, our data suggest that overexpression of INPP4B promotes NPM1-mutated leukemia cell proliferation through SGK3 activation. High levels of INPP4B are at least partially induced by the NPM1 mutant via ERK/Ets-1 signaling. Importantly, high INPP4B showed a trend towards poor outcome in NPM1-mutated patients. These findings indicate that INPP4B targeting might be a potential therapeutic strategy for NPM1-mutated AML.

## Abbreviations

AML: Acute myeloid leukemia
ATCC: American type culture collection
BM: Bone marrow
CCK8: Cell Counting Kit-8
Cdk2: Cyclin-dependent kinase 2
DNMT3A: DNA (cytosine-5-)-methyltransferase 3 alpha
DSMZ: Deutsche Sammlung von Mikroorganismen und Zellkulturen GmbH
EFS: Event free survival
ELISA: Enzyme-linked immunosorbent assay
ERK: Extracellular signal-regulated kinase
Ets-1: Erythroblastosis virus E26 oncogene homolog
FBS: Fetal bovine serum
FLT3-ITD: FMS-like tyrosine kinase 3-internal tandem duplication
HR: Hazard ratio
INPP4B: Inositol polyphosphate 4-phosphatase II
NK-AML: Normal karyotype acute myeloid leukemia
NPM1: Nucleophosmin
NPM1-mA: NPM1-mutation type A
NPM1-wt: Wild-type NPM1
OS: Overall survival
PI: Phosphoinositide
PI3K: Phosphoinositide-3 kinase
PIP: Phosphatidylinositol phosphate
PIP2: Lipid phosphatidyl-inositol bisphosphate
PIP3: Lipid phosphatidyl-inositol trisphosphate
PTEN: Phosphatase and tensin homolog
PVDF: Polyvinylidene difluoride
PX: Phox homology
qRT-PCR: Quantitative real-time PCR
RT-PCR: Reverse transcription
SDS: Sodium dodecyl sulfate
SGK3: Serum and glucocorticoid-regulated kinase-3
SHIP: Src homology 2-containing inositol phosphatase
shRNA: Lentiviral small hairpin RNA
TBS: Tris-buffered saline
TCGA: The Cancer Genome Atlas
WHO: World Health Organization
